# Stylopization by *Xenos* spp. (Xenidae, Strepsiptera) in invasive alien hornet, *Vespa velutina*, in South Korea

**DOI:** 10.1051/parasite/2025004

**Published:** 2025-02-17

**Authors:** Il-Kwon Kim, Chang-Jun Kim, Jeong-Hwan Choi, Hyun Jun Kang, Moon Bo Choi

**Affiliations:** 1 Division of Forest Biodiversity, Korea National Arboretum 11186 Pocheon Republic of Korea; 2 Division of Gardens and Education, Korea National Arboretum 11186 Pocheon Republic of Korea; 3 Haesol Eco-friendly Research Institute 46720 Busan Republic of Korea; 4 Institute of Agricultural Science and Technology, Kyungpook National University 41566 Daegu Republic of Korea; 5 Department of R&D, Wild Beei 39864 Chilgok Republic of Korea

**Keywords:** Strepsiptera, *Vespa velutina*, Invasive species, DNA barcodes, *Xenos moutoni*, *X. oxyodontes*

## Abstract

The invasive hornet *Vespa velutina* Lepeletier, which first invaded South Korea in 2003, has spread throughout the country, significantly affecting apiaries, ecosystems, and human health. *Xenos* spp. (Xenidae, Strepsiptera) are primarily parasitic to social wasps, with *V. analis* being the only known host in Korea. Until recently, no parasites or parasitoids on *V. velutina* had been discovered. In 2020, strepsipteran parasites were discovered on 11 hornet workers in Andong City, South Korea. These parasites, comprising four larvae and seven pupae, were all male, except for one individual of an undetermined sex. Molecular analysis and morphological examination identified the parasites as *Xenos moutoni* (du Buysson, 1903) and *X. oxyodontes* Nakase & Kato, 2013. This marks the first recorded instance of strepsipteran parasites on *V. velutina* in regions invaded by this hornet. Although the exact infection rate of these parasites could not be determined, it appears that native strepsipteran parasites have adapted to a non-native *Vespa* species. Stylopization, the condition caused by these parasites, is known to negatively affect hornet colonies: infected workers do not contribute to nest activities, hindering nest development, and infected reproductive individuals (males and new queens) do not mate, which impedes the establishment of new colonies. However, due to the hornet’s high reproductive rate and compensatory mechanisms, the overall control effect of the parasites is likely to be minor.

## Abstract (fr)

Le frelon invasif *Vespa velutina* Lepeletier, qui a envahi la Corée du Sud pour la première fois en 2003, s’est répandu dans tout le pays, affectant considérablement les ruchers, les écosystèmes et la santé humaine. Les espèces de *Xenos* (Xenidae, Strepsiptera) sont principalement des parasites des guêpes sociales, *V. analis* étant le seul hôte connu en Corée. Jusqu’à récemment, aucun parasite ou parasitoïde sur *V. velutina* n’avait été découvert. En 2020, des strepsiptères parasites ont été découverts sur 11 ouvrières de frelons dans la ville d’Andong, en Corée du Sud. Ces parasites, comprenant quatre larves et sept pupes, étaient tous mâles, à l’exception d’un individu de sexe indéterminé. L’analyse moléculaire et l’examen morphologique ont permis d’identifier les parasites comme étant *Xenos moutoni* (du Buysson, 1903) et *X. oxyodontes* Nakase & Kato, 2013. Il s’agit du premier cas enregistré de strepsiptères parasites chez *V. velutina* dans les régions envahies par ce frelon. Bien que le taux d’infection exact de ces parasites n’ait pas pu être déterminé, il semble que les strepsiptères parasites indigènes se soient adaptés à une espèce de *Vespa* non indigène. La stylopisation, la condition causée par ces parasites, est connue pour affecter négativement les colonies de frelons : les ouvrières infectées ne contribuent pas aux activités de nidification, ce qui entrave le développement du nid, et les individus reproducteurs infectés (mâles et nouvelles reines) ne s’accouplent pas, ce qui empêche l’établissement de nouvelles colonies. Cependant, en raison du taux de reproduction élevé du frelon et de ses mécanismes compensatoires, il est probable que l’effet global de contrôle par ces parasites ne sera que mineur.

## Introduction

The increasing invasion of invasive alien species (IAS), driven by climate change and global trade, is increasingly affecting biodiversity, human health, and ecosystems [28]. The global economic effects of IAS are estimated to reach at least US$ 1.288 trillion (2017 US dollars) in approximately 50 years [71], with invasive insects alone accounting for at least US$ 70.0 billion annually [7]. Among IAS, social wasps pose unique challenges, causing significant ecological disturbance and economic losses, particularly in the beekeeping industry [1, 4, 33]. Moreover, unlike invasive agricultural pests, social wasps present serious public health risks due to their venom, which can be lethal [11, 19, 20], leading to a rise in stinging incidents, especially in urban areas [35, 50].

Among the social wasps, *Vespa velutina* Lepeletier, 1836 (Asian hornet), native to China, first invaded Korea in 2003 [13, 37] and spread to Japan in 2013 [67]. Its first invasion in Europe was confirmed in France in 2004 [22] and has since spread to at least ten countries, including Germany, Luxembourg, and the Netherlands [27, 60]. In Europe, efforts to control *V. velutina* have involved innovative methods, such as radio tracking, radar, and traps [34, 41, 42, 66], while research on its natural enemies for biological control is ongoing.

Biological control, using natural enemies is an effective alternative to chemical control and is widely used for managing IAS [21]. The efficacy of this method depends on understanding the specific characteristics and interactions between the target IAS and its natural enemies [70]. In France, one parasite and one parasitoid of *V. velutina* have been identified: *Conops vesicularis* Linnaeus (Diptera: Conopidae) and *Pheromermis vesparum* Kaiser (Nematoda: Mermithidae), which infest the abdomen of *V. velutina* [16, 69]. Additionally, the honey buzzard, *Pernis apivorus* Linnaeus, has been recorded as a predator of wasps in Spain [43, 59].

In Korea, native social wasps have a range of natural enemies, including parasites, such as *Pyralis regalis* Denis & Schiffermüller, *Hypsopygia mauritialis* Boisduval (Lepidoptera: Pyralidae) and *Anatrachyntis japonica* Kuroko (Lepidoptera: Cosmopterigidae) [63]. The parasitoids include *Xenos* spp. (Strepsiptera: Xenidae) [44], *Bareogonalos xibeidai* Tan & van Achterberg (Hymenoptera: Trigonalyidae) [39], *Latibulus nigrinotum* Uchida and *L. flavopetiolus* Oh & Lee (Hymenoptera: Ichneumonidae) [53], *Elasmus japonicus* Ashmead, and *E. polistis* Burks (Hymenoptera: Eulophidae) [38]. Predators such as the yellow-throated marten (*Martes flavigula* Boddaert) [14] and Asiatic black bear (*Ursus thibetanus ussuricus* Heude) have also been documented [29]. In Korea, only limited natural enemies of *Vespa velutina* are known, including the predator marten [36] and parasites *P. regalis* and *H. mauritialis* [63]; however, no parasitoids have been recorded to date.

Strepsipteran parasites are intriguing candidates for biological control. These twisted-wing parasites infest hosts from seven insect orders [15]. Stylopidae and Xenidae mainly parasitize Aculeata, with Xenidae targeting various wasps such as Crabronidae, Sphecidae, and Vespidae [15, 57]. These organisms are considered parasitoids because they directly or indirectly kill the host during their development [31]; however, we have designated them as “strepsipteran parasites” in this paper to simplify the text.

Strepsipteran parasites exhibit extreme sexual dimorphism. Females, resembling grubs, never leave the host, while males leave the host and approach the female for mating. These parasites develop in the abdomen of their hosts, at the fourth instar larvae stage, the cephalothorax slightly extrudes out of the abdominal segments. The males mature as winged adults within wasp nests and approach individual wasps parasitized by neotenic females and mate before subsequently dying [3, 25].

In 2020, strepsipteran parasites were discovered in *V. velutina* in Andong City, South Korea, marking the first record of these parasites in *V. velutina* in the invasive range of this species. In this study, we aimed to identify the species of strepsipteran parasite and evaluate their potential for biological control agents against *V. velutina*.

## Material and methods

### Collection of nests and wasps

**Figure 1:**
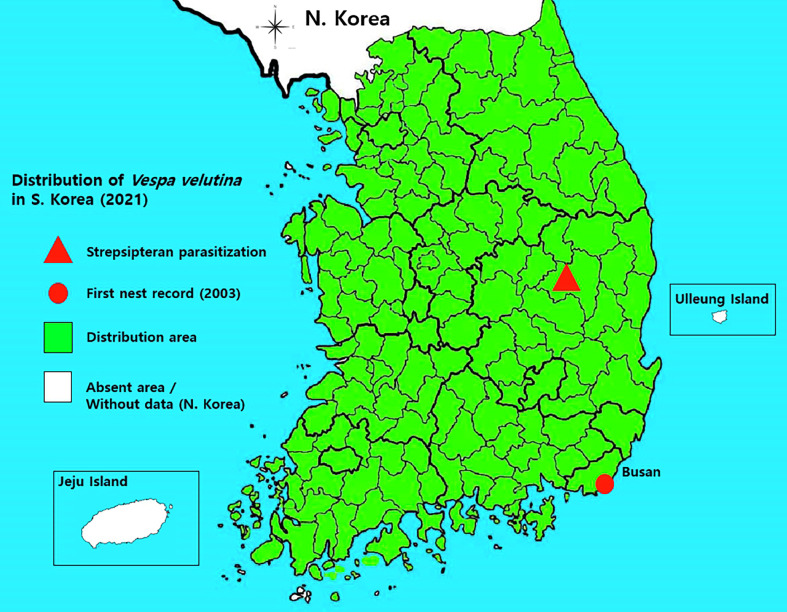
Map of the current distribution of *Vespa velutina* in South Korea and the strepsipteran parasite (red triangles).

Approximately 30 nests of *V. velutina* were collected during the fall of 2020 in Andong City, South Korea (Fig. 1). The nests are typically located 10–20 m high in trees, requiring handling to avoid destroying the nests or chasing wasps away. We applied a long-reach pruning saw, modified with a fishing pole, to cut the branches where the nests were attached, with a large mesh net placed on another fishing pole to prevent the nests from falling. If the nests were located too high to reach with the saw, and if the location was accessible by car, we hired a ladder vehicle to remove the nests safely from the trees. The collected nests were stored in large vinyl or mesh bags for transportation to the laboratory. Because the nests were collected to obtain venom from *V. velutina* individuals, all wasps were placed in an ultra-low-temperature (−80 °C) freezer to prevent deterioration of venom properties. Subsequently, poisonous females were selected from the frozen individuals. We discovered a strepsipteran parasitized in the abdomen of a few of the selected wasps by chance. Therefore, the total number of *V. velutina* parasites collected in this study was not accurately determined.

### Collection and identification of strepsipteran parasites

#### Collecting the strepsipteran parasites

**Figure 2:**
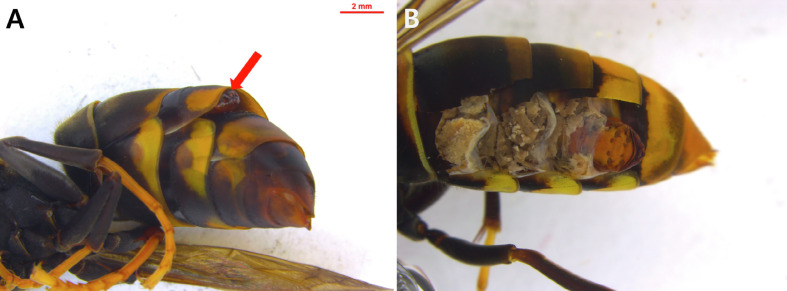
A strepsipteran parasite found in the abdomen of an adult *Vespa velutina nigrithorax*: (A) head of the parasite protruding slightly between the abdominal segments; (B) puparium inside the host abdomen.

Eleven stylopized wasps (sample No. 21-IK-V1-V11) were found among the collected *V. velutina* (Fig. 2A) and immediately frozen and stored in 99.9% ethyl alcohol. The parasites (all males) were carefully removed from the abdomens of the wasps (Fig. 2B) under a Leica stereomicroscope (Leica Microsystems, Leica M250C, Wetzlar, Germany). Generally, the sex ratio of *Xenos* spp. parasitizing wasps is higher in females [46]. However, when sorting tens of thousands of frozen wasp specimens at room temperature (approximately 20–23 °C), white frost forms on the surface of the specimens, making it harder to detect female parasites because of the flat cephalotheca sandwiched between abdominal segments. In contrast, the cephalotheca of males are relatively thick, widening the gap between the abdominal segments and making them easier to find. Among the 11 parasites, 4 were in the larval stage (21-IK-V4, V5, V6, and V10) and 7 were in the pupal stage (21-IK-V1, V2, V3, V7, V8, V9, and V11). Unfortunately, six parasite samples thawed in alcohol were destroyed (21-IK-V1, V3, V5, V9, V10, and V11) during extraction, whereas five were extracted relatively intact (21-IK-V2, V4, V6, V7, and V8).

#### Morphological identification

We attempted to morphologically identify five relatively intact strepsipteran parasites (21-IK-V2, V4, V6, V7, and V8), comprising two larvae and three pupae. The cephalotheca of the larvae remained in relatively good condition, but their bodies were very shriveled. The pupae appeared fully developed, with most body parts formed, including wings and darkened puparia, which had slightly hardened larval skin. Body parts, such as the antennae and wings, remained tightly attached, obstructing further morphological identification. However, we did identify the thoracic region. Five samples were identified according to the taxonomic keys of Nakase and Kato [52] and Benda et al*.* [5]. The cephalotheca of male larvae were imaged using a Field Emission Scanning Electron Microscope (FE-SEM, SU8220, Hitachi, Tokyo, Japan), and the whole pupal body was imaged with a Leica stereomicroscope (Leica Microsystems, Leica M250C).

#### DNA barcode sequencing

We used ten parasitic samples for DNA barcoding. To cross-validate the results of morphological identification, we analyzed the body parts of the six broken samples (21-IK-V1, V3, V5, V9, V10, and V11) along with the five samples previously identified using the taxonomic key. However, one specimen (21-IK-V5) was discarded due to extensive damage. Genomic DNA was extracted using a DNeasy Blood and Tissue Kit (QIAGEN, Manchester, UK), following the manufacturer’s protocol. We used a previously reported primer set, LCO149/HCO2198 [23, 24], for sequencing to produce a barcode region sequence of approximately 670 bp. In cases of initial sequencing failure, the mini-barcode primers MHemR1/LCO149 or MHemF1/HCO2198 [54] were also used. PCR amplification was carried out using AccuPower^®^ PCR PreMix (Bioneer, Daejeon, Korea), under the following conditions: for general barcoding, one cycle for 3 min at 94 °C, 40 cycles of 15 s at 94 °C, 30 s at 50 °C, and 40 s at 70 °C, and one cycle for 5 min at 72 °C; for mini barcoding, one cycle for 1 min at 94 °C, five cycles of 40 s at 94 °C, 40 s at 45 °C, and 1 min at 72 °C; 35 cycles of 40 s at 94 °C, 40 s at 51 °C, and 1 min at 72 °C; and one cycle of 5 min at 72 °C. PCR product purification and sequencing were conducted by Macrogen, Inc. (Seoul, Republic of Korea).

#### Sequence analysis for identification and species delimitation

The nucleotide sequences were aligned using Geneious Prime ver. 2021.1.1 [32], and BLAST searched to determine any possible taxonomic group, such as a genus or family, within Strepsiptera. The sequences of other species were mined from the NCBI database for analysis, as suggested by the BLAST search results. As applied by [24, 54], a neighbor-joining analysis (with 1000 bootstrap values) was run with COI sequences, including other species in the same genus obtained from NCBI, to identify the targeted strepsipteran species from *V*. *velutina.*

A species delimitation method, Assemble Species by Automatic Partitioning (ASAP), was performed to estimate the number of molecular operational taxonomic units (MOTUs) from the dataset, following Puillandre et al*.* [58]. ASAP analysis was run under Jukes-Cantor (JC69), Kimura (K80), and Simple Distance (SD) substitution models using the web interface (https://bioinfo.mnhn.fr/abi/public/asap/asapweb.html).

## Results

### Identification of the strepsipteran parasites using morphological characters

**Figure 3:**
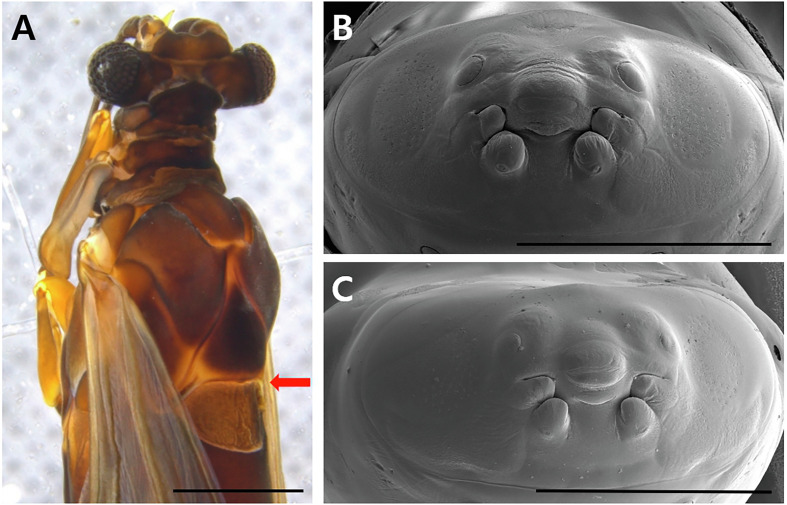
Males of *Xenos moutoni* and *Xenos oxyodontes* extracted from the abdomen of Vespa *velutina nigrithorax*: (A) almost fully-developed pupae of *X. moutoni*, 21-IK-V8; (B) cephalotheca of *X. moutoni* larvae, 21-IK-V4; (C) cephalotheca of *X. oxyodontes* larvae, 21-IK-V6. Scale bar = 1 mm.

Of the five samples, all three pupae were identified as *Xenos moutoni* (du Buysson, 1903) (21-IK-V2, V7, V8) (Fig. 3A), and the two larvae were identified as *X. moutoni* (21-IK-V4) (Fig. 3B) and *X. oxyodontes* [52] (21-IK-V6) (Fig. 3C), respectively.

### Sequencing and identification of the strepsipteran parasites using barcode sequence data

**Figure 4:**
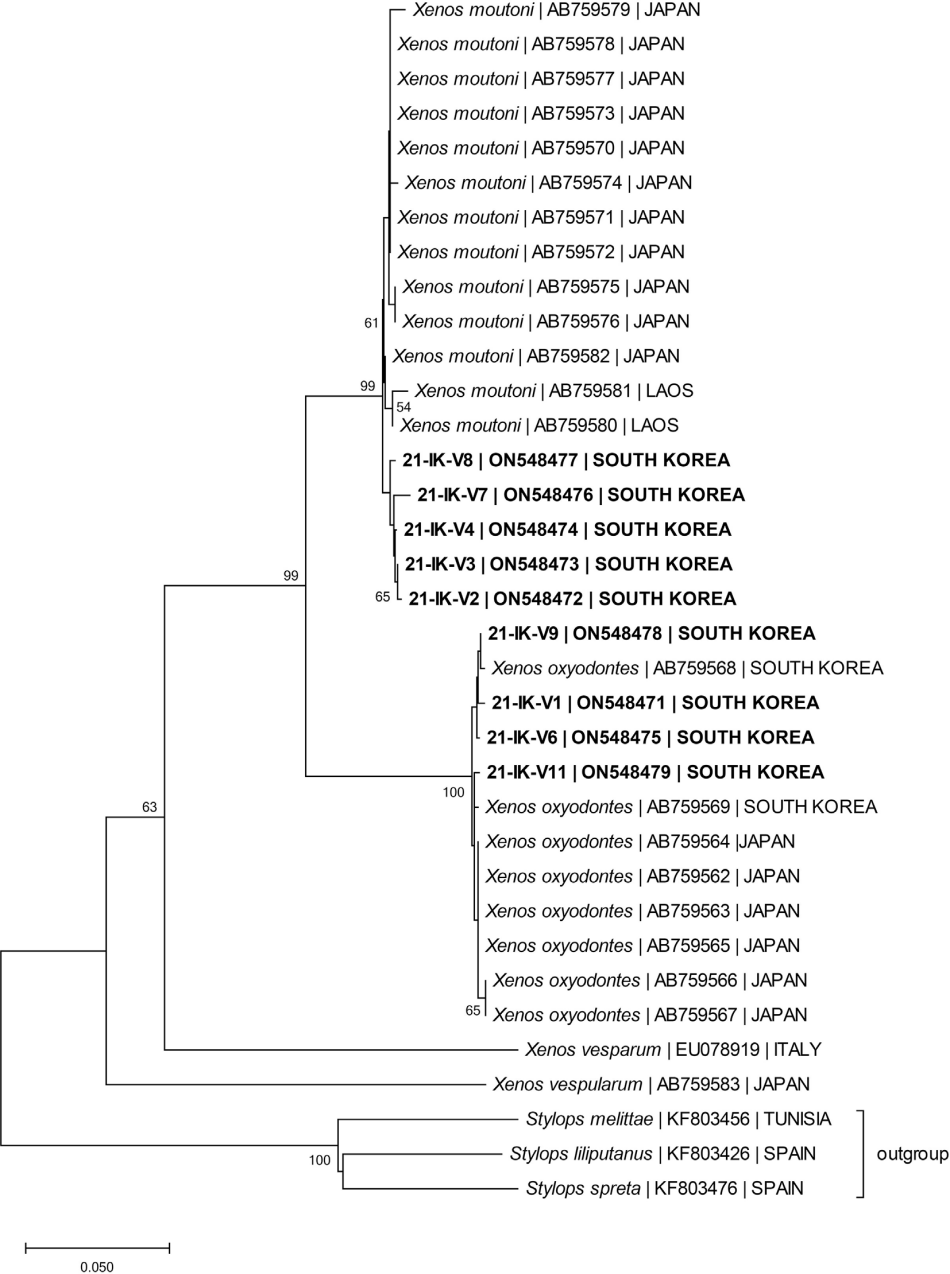
Neighbor-joining tree (with 1000 bootstrap values) inferred from the barcode data with an uncorrected P-distance. Bootstrap values are shown for the nodes. Outgroup: *Stylops melittae*, *S*. *liliputanus,* and *S*. *spreta*. Unnamed species in light blue indicate the target species of the present study.

Barcode sequencing was used to identify the strepsipteran species. Full barcode region sequencing, which normally produces approximately 600–700 bp in length, was unsuccessful. In contrast, mini-barcoding yielded sequences of mostly 300 bp from nine samples, except for 21-IK-V10, due to sequencing failure (Table 1). An initial BLAST search showed that our samples were closely grouped with *Xenos* species in Xenidae. Using the sequences of our samples and other *Xenos* species [52], the neighbor-joining analysis produced a robust tree indicating the targeted species composed of two *Xenos* species, namely *X*. *moutoni* [8] and *X*. *oxyodontes* [52] (Fig. 4).

**Table 1:** Samples from the present study and NCBI COI sequences used to identify the parasites in *Vespa velutina nigrithorax*. 21-IK-V5 and V10 were excluded due to sample destruction and sequencing failure.

Species name/sample code	NCBI accession No.	Locality	Reference
21-IK-V1	ON548471	S. Korea	The present study
21-IK-V2	ON548472	S. Korea	″
21-IK-V3	ON548473	S. Korea	″
21-IK-V4	ON548474	S. Korea	″
21-IK-V6	ON548475	S. Korea	″
21-IK-V7	ON548476	S. Korea	″
21-IK-V8	ON548477	S. Korea	″
21-IK-V9	ON548478	S. Korea	″
21-IK-V11	ON548479	S. Korea	″
*Xenos moutoni*	AB759570	Japan	[52]
	AB759571	Japan	″
	AB759572	Japan	″
	AB759573	Japan	″
	AB759574	Japan	″
	AB759575	Japan	″
	AB759576	Japan	″
	AB759577	Japan	″
	AB759578	Japan	″
	AB759579	Japan	″
	AB759580	Laos	″
	AB759581	Laos	″
	AB759582	Japan	″
*Xenos oxyodontes*	AB759562	Japan	″
	AB759563	Japan	″
	AB759564	Japan	″
	AB759565	Japan	″
	AB759566	Japan	″
	AB759567	Japan	″
	AB759568	S. Korea	″
	AB759569	S. Korea	″
*Xenos vesparum*	EU078919	Italy	[30]
*Xenos vespularum*	AB759583	Japan	[52]
*Stylops melittae*	KF803456	Tunisia	[68]
*Stylops liliputanus*	KF803426	Spain	″
*Stylops spreta*	KF803476	Spain	″

The tree topology was consistent with the maximum-likelihood tree of Nakase and Kato [52], with two large clades of *X. moutoni* and *X oxyodontes*. These two clades Interspecific Kimura-2-parameter (K2P) values among the *Xenos* species ranged from 0.0912 to 0.3397, with an average of 0.2512 among the species (Table 2). Specifically, the value between *X*. *moutoni* and *X*. *oxyodontes* was 0.0912, and between *X. hamiltoni* and *X. pecki* was 0.0448, the lowest in the dataset, while the others were higher. The interclade K2P values among the *Xenos* species categorized by country ranged from 0.0057 to 0.3397, with an average of 0.2145 among the clades (Table 3). In particular, the values among *X*. *moutoni* JAPAN, *X*. *moutoni* LAOS, and *X*. *moutoni* SOUTH KOREA were 0.0108, 0.0119, 0.0130, and the value between *X*. *oxyodontes* JAPAN and *X*. *oxyodontes* SOUTH KOREA was 0.0057, which was the lowest in the dataset, while the rest were higher. The intraspecific K2P values among the *Xenos* MOTUs ranged from 0.0000 to 0.3679, with an average of 0.1143 among the MOTUs. The average, minimum, and maximum K2P values within the three species, *X*. *moutoni*, *X*. *oxyodontes,* and *X*. *vesparum,* which form a clade, are shown in Table 4.

**Table 2:** Interspecific K2P values of Barcode region sequences from the species of Xenidae.

	1	2	3	4	5	6	7
1. Outgroup							
2. *Xenos hamiltoni*	0.2972						
3. *Xenos moutoni*	0.2939	0.1893					
4. *Xenos oxyodontes*	0.3248	0.1831	0.0912				
5. *Xenos pecki*	0.3203	0.0448	0.2017	0.2072			
6. *Xenos* sp.	0.3206	0.2543	0.2240	0.2448	0.2503		
7. *Xenos vesparum*	0.3397	0.2695	0.2383	0.2574	0.2832	0.2548	
8. *Xenos vespularum*	0.3097	0.2585	0.2462	0.2453	0.2823	0.3098	0.2911

**Table 3:** Interclade Kimura-2-parameter (K2P) values of Barcode region sequences from the species of Xenidae that were categorized by the countries.

	1	2	3	4	5	6	7	8	9	10
1. Outgroup										
2. *Xenos hamiltoni*	0.2972									
3. *Xenos moutoni* JAPAN	0.2885	0.1848								
4. *Xenos moutoni* LAOS	0.2956	0.1839	0.0108							
5. *Xenos moutoni* S. KOREA	0.3053	0.2016	0.0119	0.0130						
6. *Xenos oxyodontes* JAPAN	0.3145	0.1817	0.0910	0.0936	0.0912					
7. *Xenos oxyodontes* S. KOREA	0.3351	0.1844	0.0908	0.0920	0.0911	0.0057				
8. *Xenos pecki*	0.3203	0.0448	0.1956	0.1967	0.2170	0.2005	0.2138			
9. *Xenos* sp.	0.3206	0.2543	0.2299	0.2279	0.2094	0.2483	0.2412	0.2503		
10. *Xenos vesparum*	0.3397	0.2695	0.2353	0.2383	0.2449	0.2513	0.2635	0.2832	0.2548	
11. *Xenos vespularum*	0.3097	0.2585	0.2493	0.2581	0.2347	0.2452	0.2453	0.2823	0.3098	0.2911

**Table 4:** Intraspecific Kimura-2-parameter (K2P) values of Barcode region sequences from the species of Xenidae.

Comparisons	Mean	Min.	Max.
*Xenos moutoni*	0.0080	0.0000	0.0214
*Xenos oxyodontes*	0.0043	0.0000	0.0090
*Xenos vesparum*	0.0048	0.0031	0.0076

We conducted an ASAP delimitation analysis to investigate intraspecific partitioning using three substitution models, JC69, K80, and SD. The species-partitioning results were nearly identical across the three models (Fig. 4). In particular, for both *X*. *moutoni* and *X*. *oxyodontes*, our analyses consistently indicated that each of them was a single species. The distance values in the threshold analysis for pairwise distances grouped each taxon into a single species.

## Discussion

### Occurrence of *Xenos* spp. in South Korea

Nine species of Xenidae have been recorded in Korea [10], of which two have been identified as *X. moutoni* and *X. oxyodontes* [45, 52].

According to the Japanese literature, which has a similar Vespidae fauna to that of Korea, most *Vespa* species inhabiting Japan are parasitized by strepsipteran species [44, 46, 49, 65].

However, a record of strepsipteran parasites has been reported only in *V. analis* Fabricius using bait traps in Andong City, Korea [45]. Coincidentally, a previously reported study area [44] and this study area overlapped with Andong (Fig. 1); however, this area is not a specific area in the study of strepsipteran species, as they are found throughout South Korea.

Only a few studies have reported strepsipteran parasites in Korea, despite ten species (including subspecies) of *Vespa* being recorded [12]. Notably, strepsipteran parasites appeared in 6–7 Korean *Vespa* specimens collected by another group of researchers (unpublished data; MB Choi, pers. comm.). Therefore, in Korea, strepsipteran parasites in *Vespa* species are not rare, occurring in a specific area, but rather appear to be widespread. This study is the first to record strepsipteran parasitism in the invasive hornet *V. velutina*.

A recent record of parasitism by *X. moutoni* and *X. yangi* in China, the native region of *V. velutina*, has been reported [17, 72]. However, no cases of parasitism have been reported in the regions where it has invaded. Therefore, the discovery of parasitoids on the invasive hornet, *V. velutina nigrithorax,* may have implications for the development of potential biological control agents in the future.

*Vespa velutina* first appeared in Andong in 2010 [12, 37]. As nearly ten years have passed since the invasion of this area, the strepsipteran parasite appears to have already spread to the lower southern areas. In addition, most of the *X. moutoni* and *X. oxyodontes* discovered in this study used *Vespa* species as hosts; therefore, we speculated that *X. vespularum* Kifune & Maeta, *X. vesparum* Rossi, and new species may be discovered if *Vespula* and Polistinae are further investigated [6].

### Species delimitation

Nakase and Kato [52] reported a molecular phylogenetic tree for *X*. *moutoni* and *X*. *oxyodontes*, suggesting that *X*. *moutoni* may form a species complex. In this study, we conducted a molecular phylogenetic analysis of *Xenos* spp., focusing on *X*. *moutoni* and *X*. *oxyodontes*, and constructed a phylogenetic tree. Although the MOTUs of *X*. *oxyodontes* formed a weak subclade, they did not show significant differences. In contrast, *X*. *moutoni* clearly showed subclades in South Korea, Japan, and Laos, suggesting the potential formation of species complexes, as shown in the tree by Nakase and Kato [52]. However, the small initial sample size and underdevelopment of some samples made it difficult to confirm morphological traits. Because we used the entire sample for DNA extraction, external morphological taxonomy could not be applied. To address this, we used a species delimitation analysis based on the COI barcode region to confirm the boundaries between species and MOTUs as an alternative method for species partitioning. ASAP, which was recently developed based on pairwise distances, was conducted using the following nucleotide substitution models: JC69, K80, and SD. The pairwise distance values among the MOTUs of *X*. *moutoni* and *X*. *oxyodontes* in each model were all lower than the threshold values for species delimitation calculated by ASAP analysis, indicating that they are unlikely to be different species (Table 4, Fig. 4). For a detailed taxonomic investigation into the formation of regional subclades, further sampling of specimens from each region and examination of the external morphology of fully developed adult specimens are necessary to conduct thorough analyses at both the morphological and molecular levels.

### Particularities of *Xenos* parasitism

In social insects, such as honeybees and ants, colony collapse often occurs due to infection by natural enemies, including parasitoids, viruses, mites, and fungi [2, 9, 40, 62].

Unfortunately, colony collapse by parasites or parasitoids in social wasps is rare; therefore, the effectiveness of biological control against invasive social wasps is minimal. For example, the control effects of the parasitoid *Sphecophaga vesparum* Curtis against the invasive *Vespula* species, and the endoparasitoid nematodes *Pheromermis vesparum* and *Conops vesicularis* against *V. velutina* were insignificant [4, 16, 69].

*Xenos* species are the most common parasitoids of social wasps and their parasitic mechanisms on wasps are unique. *Xenos* species mainly stylopize workers, males, and new queens, except for the foundress. Except for males, among stylopized wasps in autumn, females, such as new queens and workers, go into hibernation [65]. In particular, stylopized workers live longer than non-stylopized workers. Hence, unlike healthy workers, they overwinter and are often found with the foundress in the tree sap the following year’s spring [3].

The stylopized females emerge from hibernation at the end of April and survive until early July while searching for tree sap. At this time, the eggs of *Xenos* species in the stylopized female body hatch and crawl out as first-instar larvae, which fall off from the female body when the females eat the tree’s sap. Females die as soon as all *Xenos* larvae escape [49].

According to Matsuura and Yamane [49], approximately 1000–2000 *Xenos* larvae are generated daily by *V. mandarinia* workers (max. 2362), resulting in 29,843–36,844 *Xenos* larvae per individual (average: 34,581).

After escaping from the host at the tree sap point, they wait for other wasps (hosts) to visit, attach to their bodies, and move to each nest to parasitize. The *Xenos* larvae that invade the nest parasitize the larvae of each wasp in this manner [26, 48].

The infection rate of parasites is positively correlated with the host group size [55]. Therefore, the infection rate of wasps by *Xenos* species may increase as the number of visits to the tree sap point increases, which means that the larger the colony, the greater the number of wasps exposed to *Xenos* species at the tree sap point. This may increase the infection rate. Colonies of the invasive hornet *V. velutina* are larger than all Korean *Vespa* species [13, 61]. Although the exact rate of infection is unknown, it seems that the parasitoids of *Xenos* species will continue to appear approximately 20 years after the invasion of *V. velutina*.

### Development of potential control agents

Wasps stylopized by *Xenos* species are not killed by them, but exhibit unusual behavior. Stylopized workers are largely inactive, occasionally showcasing minor behaviors such as fanning, patrol, prey malaxation, and food exchange with larvae. They do not engage in critical activities for colony development, such as nest construction, defense against natural enemies, and larval rearing [49]. Therefore, stylopized workers are a major hindrance to nesting and colony expansion. Additionally, a higher ratio of stylopized workers has been shown to correlate with smaller nest sizes [47, 51, 64], a phenomenon particularly detrimental to early colonies with few workers [47]. The proper expansion of the nest relies heavily on worker activity; thus, an abundance of stylopized workers leads to poor larval rearing and stunted nest growth, resulting in colony failure or underdeveloped nests [47].

In Japan, the parasitism rate of *Vespa* species is reported to be approximately 0.8–10.6% per individual and 23.5–48.2% per nest, indicating that while the parasitism rate per individual is relatively low, the rate per nest is quite high [46, 47, 49]. Although parasitism rates are highly variable according to the study area, method (trap use), and period, and most Japanese studies have focused on *V. analis*, this parasitic rate is also prevalent in other *Vespa* species.

Therefore, the emergence of these parasitoids is encouraging in invasive hornet management. Although there is a limit to the direct control effect of these parasitoids, the manager’s role in indirectly reducing the dominance and increase of invasive hornets over a long period through host-parasite evolutionary interactions seems possible [18, 56].

However, in terms of a more direct control role, *Xenos* species exhibit high parasitic rates among workers from June to August, with increased parasitism rates for reproductive wasps (new queens and males) during the autumn reproductive period. For example, in *V. analis*, 1.3–6.4% of males were stylopized in the fall, and 9–48% of overwintered females were stylopized in the following spring [44–47].

Males or new queens lose fertility when stylopized by *Xenos* species [47, 65]. During the reproductive period, stylopized males in the nest do not respond to mating activity, reducing the mating rate of new queens, and stylopized new queens hibernate without mating with the males [49]. Stylopized queens from hibernation roam the tree sap spots until June–July, when they die. Therefore, the control effect occurs because the parasitism of reproductive individuals negatively affects the development of their colonies [3, 44, 46, 47]. However, despite these negative effects, parasitism does not drastically reduce hornet colony strength because of the high reproductive efficiency of *Vespa* colonies, supported by a density-dependent compensation mechanism [69].

In conclusion, the most prevalent and highest infection rate among parasites of social wasps was stylopization by *Xenos* spp. Although this parasitism may have some negative effects, the control effect is minimal; this can be applied to native and invasive wasps.
